# Feasibility of an Intervention Delivered via Mobile Phone and Internet to Improve the Continuity of Care in Schizophrenia: A Randomized Controlled Pilot Study

**DOI:** 10.3390/ijerph182312391

**Published:** 2021-11-25

**Authors:** Christina Gallinat, Markus Moessner, Sandra Apondo, Philipp A. Thomann, Sabine C. Herpertz, Stephanie Bauer

**Affiliations:** 1 Center for Psychotherapy Research, University Hospital Heidelberg, 69115 Heidelberg, Germany; markus.moessner@med.uni-heidelberg.de (M.M.); stephanie.bauer@med.uni-heidelberg.de (S.B.); 2 Department of General Psychiatry, University Hospital Heidelberg, 69115 Heidelberg, Germany; sandra.apondo@med.uni-heidelberg.de (S.A.); sabine.herpertz@med.uni-heidelberg.de (S.C.H.); 3 Zentrum für Seelische Gesundheit, Gesundheitszentrum Odenwaldkreis GmbH, 64711 Erbach im Odenwald, Germany; philipp.thomann@gz-odw.de

**Keywords:** schizophrenia, aftercare, relapse prevention, internet, mobile, digital

## Abstract

Schizophrenia is a severe mental illness associated with a heavy symptom burden and high relapse rates. Digital interventions are increasingly suggested as means to facilitate continuity of care, relapse prevention, and long-term disease management for schizophrenia spectrum disorders. In order to investigate the feasibility of a mobile and internet-based aftercare program, a 2-arm randomized controlled pilot study was conducted. The program could be used by patients for six months after inpatient treatment and included psychoeducation, an individual crisis plan, optional counseling via internet chat or phone and a supportive monitoring module. Due to the slow pace of enrollment, recruitment was stopped before the planned sample size was achieved. Reasons for the high exclusion rate during recruitment were analyzed as well as attitudes, satisfaction, and utilization of the program by study participants. The data of 25 randomized patients suggest overall positive attitudes towards the program, high user satisfaction and good adherence to the monitoring module. Overall, the results indicate that the digital program might be suitable to provide support following discharge from intensive care. In addition, the study provides insights into specific barriers to recruitment which may inform future research in the field of digital interventions for severe mental illness.

## 1. Introduction

Schizophrenia is a severe psychiatric illness affecting approximately 0.3–0.7% of the population throughout their lifespan. The disorder is characterized by positive and negative symptoms covering a heterogenous range of dysfunctions in behavior, thoughts, perception and affect [1]. Schizophrenia is recognized as a chronic and disabling illness, which is listed as one of the 20 leading causes of disability worldwide [2,3]. Various studies have shown that schizophrenia is associated with high relapse rates of up to 65% within three years and up to 82% within five years after the first psychotic episode [4,5]. Consequently, relapse prevention and long-term disease management are of the highest importance. A key element in sustaining remission after the first psychotic episode is a maintenance therapy with antipsychotic medication. Withdrawal of medication has been identified as a strong predictor for the recurrence of psychotic symptoms in numerous studies [6,7,8]. Therefore, antipsychotic medication should be maintained for at least two years after a first single psychotic episode [9]. Furthermore, cognitive behavior therapy is also recommended for relapse prevention [10]. To ensure early detection of warning signs as well as to improve medication adherence, close contact to personal mental health professionals is crucial. Digital interventions have been discussed as promising means in this context as they allow professionals to maintain contact with patients over the long-term and may serve as easily accessible and low-threshold sources of support.

Internet-based and mobile interventions have been shown to be effective for the self-management and treatment of a variety of mental disorders, e.g., [11,12]. Concerning the fields of aftercare, relapse prevention, and long-term disease management, there is also evidence for the potential of digital interventions, but clearly more studies are needed [13]. For schizophrenia spectrum disorders, various internet-based and mobile interventions have been developed and evaluated over the past decade. These include rather specific interventions focusing, for example, on emotion recognition or motivation [14,15], as well as programs targeting general improvement of symptoms, detection of early warning signs, medication adherence and relapse prevention [16,17,18]. A systematic review on digital interventions for psychosis from 2014 concluded that such interventions are feasible and acceptable for patients with psychosis [19]. In terms of intervention efficacy, the review reports positive outcome effects in some studies (e.g., medication adherence, psychotic symptoms), but it also points out the heterogeneity of studies and the lack of high-quality studies. Since then, several additional studies have been conducted on this topic: In a large 2-arm RCT with *N* = 1139 participants, Välimäki et al. [17] investigated a 12-month text message intervention. The results did not support the efficacy of the intervention in terms of the rate of re-hospitalizations or time between hospitalizations. In contrast, Xu et al. [20] (*N* = 278) reported, in a 2-arm randomized controlled trial, an improved medication adherence as well as reduced rates of relapses and re-hospitalization for an intervention, which involved support by lay health supporters (e.g., someone from the patient’s family) and mobile texting for medication reminders, health education and monitoring of early signs of relapse. However, no effects on the symptom status were found. Another RCT investigated a 12-month intervention including medication reminders, feedback messages, cognitive training and optional televisits [18]. In a sample of 290 patients with paranoid schizophrenia, no differences were found regarding hospitalizations, length of hospitalizations and improvement of the general clinical status, but participants in the intervention group had lower affective symptoms at post assessment compared with the control group. Recently, Westermann et al. [21] evaluated an 8-week, CBT-based self-help intervention (including e.g., information modules, textual worksheets, audio files, exercises, personal feedback) in 101 patients with schizophrenia spectrum disorders. The RCT revealed a statistically significant decrease of self-reported hallucinations in the intervention group compared with the waitlist control group. However, the results did not show any intervention effects on clinician-rated positive and negative symptoms nor on self-reported depression and worrying.

In summary, the heterogeneity of studies in terms of healthcare settings, interventions, outcome measures and study procedures impedes an overall evaluation of the potential of digital interventions for schizophrenia spectrum disorders to date. To our knowledge, no digital intervention specifically designed for the aftercare or relapse prevention in schizophrenia spectrum disorders is currently available in German. Therefore, we developed the internet-based and mobile intervention “HEINS” to enhance the continuity of care for individuals with schizophrenia spectrum disorders following inpatient psychiatric treatment. In this paper, we report on a pilot study that we conducted in order to investigate patients’ willingness to participate, their attitudes towards the digital intervention, user satisfaction, and program utilization as well as the feasibility of study procedures.

## 2. Materials and Methods

### 2.1. Inclusion and Exclusion Criteria

Participants were eligible for participation if they had a primary diagnosis of a schizophrenia spectrum disorder (F20–F29; World Health Organization (WHO)) [22] and if they completed inpatient treatment at the hospital for General Psychiatry at the University Hospital Heidelberg. Further inclusion criteria were a minimum age of 18, sufficient German language skills, possession of a mobile phone, internet access, and the ability to give informed consent. Also, patients needed to have an outpatient psychiatric provider following discharge from the hospital. Exclusion criteria were substance addiction (besides tobacco) in the last two years, traumatic brain injury in the past, and an acute psychotic disorder (duration less than four weeks).

### 2.2. Study Design and Procedures

The present study followed a 2-arm randomized controlled design including an intervention group (treatment as usual plus HEINS) and a control group (treatment as usual). Recruitment aimed for a sample size of 50 participants (25 per group) and was conducted at the hospital for General Psychiatry at the University Hospital Heidelberg. In close contact with the clinical staff, the research team assessed the eligibility of patients and informed eligible patients about the study.

Eligible patients who were interested in participating, were asked to give written informed consent and to answer a short questionnaire regarding their attitudes and expectations towards the digital aftercare intervention HEINS. At discharge from the hospital, a baseline interview was conducted. At the end of the interview the patient was informed about the study group he or she had been randomly assigned to. Participants of the intervention group then directly received an introduction into the program and the interviewer and the patient prepared a personal crisis plan together and entered it into the software. Participants of the intervention group were then able to use the HEINS program for six months as an add-on to treatment as usual. The control group received treatment as usual only and did not further interact with the study staff. Both groups were invited for the post assessment six months after discharge from inpatient treatment. Patients who were eligible but had refused participation, were invited to answer a short questionnaire assessing reasons for their unwillingness to participate. The study was approved by the ethics committee of the Medical Faculty of Heidelberg University and registered at the ISRCTN registry (ISRCTN15399617).

### 2.3. Measures

#### 2.3.1. Sociodemographics and Clinical Variables

Sociodemographic information, diagnoses, duration of illness, discharge medication, and the number of former inpatient treatments because of schizophrenia were assessed within a short questionnaire, completed by a psychiatrist at the hospital.

#### 2.3.2. Psychopathology

The current symptom status was assessed with the Positive and Negative Symptom Scale (PANSS) [23], which is considered the gold standard for the assessment of multidimensional symptoms in schizophrenia spectrum disorders. The scale consists of 30 items, which are rated on a 7-point Likert scale from 1 (“absent”) to 7 (“extreme”). Accordingly, the PANSS total score ranges between 30 and 210. Positive symptoms and negative items are assessed with seven items each and 16 items refer to global symptoms (e.g., depression, tension, disorientation). A PANSS score of 58 corresponds to a “mildly ill” status, 75 to “moderately ill”, 95 to “markedly ill” status and 116 to a “severely ill” status according to Leucht et al. [24]. In the present study, the PANSS was completed on the basis of the baseline interview which was conducted just before or right after discharge from the hospital, and six months after discharge.

#### 2.3.3. Attitudes and Expectations

Attitudes and expectations towards the HEINS intervention were measured with 21 items, which were rated on a 4-point Likert scale from 1 (“not at all”/”definitely no”) to 4 (“very”/”definitely yes”). This questionnaire was completed by all study participants prior to randomization.

#### 2.3.4. User Satisfaction and Program Utilization

User satisfaction of participants in the intervention group was investigated with 24 items assessing personal benefit (Likert scale 1 (“not at all”) to 4 (“very”)) and satisfaction with the program modules (Likert scale 1 (“does not apply at all”) to 4 (“applies exactly”) and “not able to evaluate”). Moreover, participants were asked about the adequacy of the program, its duration, the frequency of the monitoring, whether they would recommend it to others, and their overall satisfaction. Program utilization was automatically recorded within the program (e.g., number of completed monitoring assessments, booked chat appointments).

#### 2.3.5. Reasons for Declining Participation

Patients, who were not willing to participate in the study, were asked to complete a short questionnaire regarding reasons for non-participation. Reasons were assessed with 13 items, rated on a 3-point Likert scale from 1 (“not important at all”) to 3 (“very important”).

### 2.4. Intervention

The “Heidelberg internet-based aftercare for patients with schizophrenia spectrum disorders” (HEINS) intervention was developed using the “Assessment and monitoring of mental health” (ASMO) software [25]. HEINS consists of several modules, which patients could use as an add-on to conventional care for six months following their inpatient treatment; 1, psychoeducation on schizophrenia (e.g., symptoms, treatment, early warning signs); 2, access to an individual crisis plan (prepared at baseline, accessible via the HEINS website); 3, personal contact with a psychiatrist from the inpatient treatment unit via internet chat and telephone; and 4, weekly supportive monitoring.

The supportive monitoring module served as the central module of the program. Once a week, participants were invited via short message service (SMS) to answer a short monitoring questionnaire assessing general wellbeing, amount of sleep, social contacts, medication adherence and anxiety/insecurity in the last seven days. Entries could be made via SMS or via smartphone/internet. In response to their answers, participants immediately received an automated, tailored feedback message aiming at the reinforcement of positive states and the interruption of negative developments by suggesting concrete behavioral changes (e.g., “It’s good that you are regularly taking your medication. At the same time, it seems that you are not feeling well. If that doesn’t change, you should seek further support (e.g., from your outpatient psychiatrist or via chat/phone within HEINS”). If the monitoring was not answered within 24 h, an automated reminder message was sent. In case of three unanswered monitoring questionnaires in a row or pre-defined critical entries in the monitoring, the study clinician received a notification (“alarm message”) and called the participant as soon as possible to clarify, if the patient was in need for further professional support. This alarm procedure was triggered by the following monitoring entries: medication stopped, medication reduced without medical advice, slept less than four hours on average in the last week and anxiety/insecurity at minimum three days in the last week. The weekly supportive monitoring is expected to enhance self-management skills regarding crucial aspects for the course of illness (e.g., medication adherence, sleep). Furthermore, the described alarm procedure allows the early recognition of warning signs and the timely initiation of further support if needed.

### 2.5. Statistical Analysis

Data on attitudes and expectations, reasons for non-participation as well as program use and user satisfaction were analyzed by means of descriptive statistics. Continuous variables regarding attitudes, expectations and user satisfaction were dichotomized by splitting 4-point Likert scales in the middle (e.g., Disagree: “definitely no”, “rather no”; Agree: “rather yes”, “definitely yes”). Data on these variables were then analyzed with frequencies. Changes in symptomatology are reported on a descriptive level, since the small sample size did not allow sufficiently powered efficacy analyses. Data analyses were conducted with IBM® SPSS® Statistics 25.0 (SPSS, Inc., Chicago, IL, USA).

## 3. Results

### 3.1. Recruitment and Participant Flow

The present study could not be completed according to the study plan, since the enrollment rate lagged substantially behind the time schedule. Therefore, recruitment was stopped before the planned sample size was achieved, suggesting that the trial was not feasible in its original setup.

**Figure 1:**
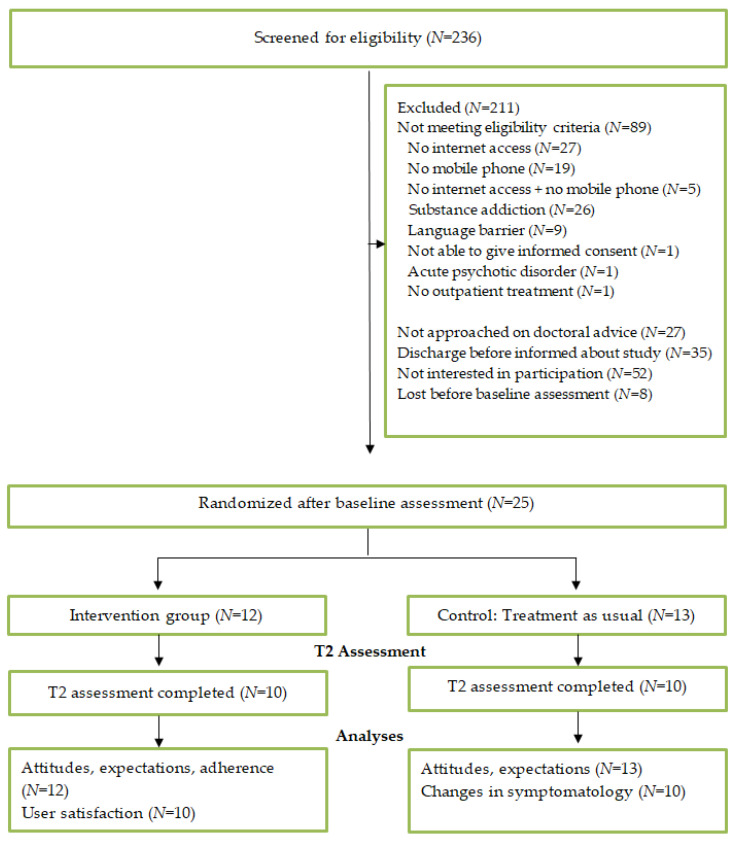
Participant flow.

Participants were recruited over a period of 29 months. During this period, 236 patients with diagnoses in the schizophrenia spectrum (F20–F29), who received inpatient treatment in the General Psychiatry of the University Hospital Heidelberg and were at least 18 years old, were screened for eligibility. Out of these, 89 participants (38%) were excluded because they did not meet the criteria (Figure 1). This was most commonly because patients had no internet access, no mobile phone, or neither of them (*N* = 51; 22% of all screened patients). Twenty-six patients (11%) had to be excluded due to substance addiction and nine (4%) due to insufficient German language skills. Twenty-seven patients (11%) were not approached following doctoral advice for several reasons. Such reasons included that the patients were too severely impaired (e.g., very high depressive symptoms, cognitive impairment), had no insight or acceptance of their disease or were labeled too uncooperative to be approached for a study. One person was not approached since mobile phones were a central component in her delusions.

Moreover, 35 patients (15%) did not undergo further screening for eligibility due to preterm discharge or organizational reasons which meant that the patient could not be approached before discharge. Organizational problems mainly included difficulties in communication procedures (e.g., no timely notification when a patient is about to be discharged), which were often caused by changes in clinical staff. In total, 85 patients were invited to participate in the study. Out of those, 61% (*N* = 52) declined to participate. 39% (*N* = 33) were interested in participation, but eight of them did not enter the study (e.g., because they changed their mind or did not respond to invitations for the baseline assessment). Thus, only 25 patients were randomized to the intervention and control group respectively.

### 3.2. Participants

Twenty-five patients gave informed consent and underwent the baseline assessment, so that they were randomly assigned to one of the study groups. The total sample consisted of 16 women (64.0%) and 9 men (36.0%), and the mean age was 36.3 years (SD = 12.23; range 18–64). The average duration of the schizophrenia spectrum disorders was 7.7 years (SD = 7.45; range 0–20) and participants underwent 2.9 previous inpatient treatments due to the disorder on average (SD = 3.59; range 0–13). At baseline, i.e., at discharge from inpatient treatment the sample had a mean PANSS total score of 47.21 (SD = 14.52; range 33–87), which falls below the benchmark of 58 points marking a “mildly ill” status according to Leucht et al. [24]. In detail, 10 patients had total scores between 33 and 40, and 8 patients had total scores between 41 and 58. Four patients fell into the category of being moderately ill (59–75) and only one patient had a score of 87 (markedly ill). A detailed overview of demographic and clinical variables per group is provided in Table 1. Both groups show a marginally lowered mean PANSS total score at the 6-months assessment.

### 3.3. Reasons for Declining Participation

Twenty-four out of the 52 patients who declined participation completed the questionnaire assessing reasons for non-participation. Participants, who did not answer this questionnaire, were asked orally for their main reasons not to participate. This way, 25 patients provided reasons for their decision. Thus, in total 49 out of 52 (94.2%) patients gave information about their reasons to decline study participation. Most commonly mentioned reasons were the impression that there was “no need because of sufficient aftercare support” (57.5%, *N* = 27), “no interest” (46.8%, *N* = 22), “no time to participate” (34.0%, *N* = 16), “not expecting the intervention to be helpful” (34.0%, *N* = 16), “too much effort expected by the intervention” (29.8%, *N* = 14) and “too much effort caused by the study” (27.7%, *N* = 13). Other reasons were concerns about personal skills regarding internet and mobile phones (20.4%, *N* = 10), as well as concerns about data privacy (18.4%, *N* = 9) and feeling controlled (18.4%, *N* = 9). Percentages refer to the sample of 49 individuals, who provided data about reasons for not participating. Multiple responses were possible.

### 3.4. Attitudes and Expectations

Attitudes and expectations assessed prior to randomization were overall positive (Table 2). Most participants (70 to 100%) indicated that they would use the different modules of the HEINS program, especially the supportive monitoring module. Moreover, participants expected positive effects on their well-being and that the program would provide a feeling of security and support after discharge.

### 3.5. User Satisfaction

Overall, participants in the intervention group (*N* = 10) were very satisfied with the program. All participants indicated that the HEINS program is an appropriate aftercare support for patients with schizophrenia spectrum disorders and 8 participants would recommend the program to a friend in the same situation. Furthermore, 9 participants indicated that they would participate again and that they were satisfied overall with the program. Seven participants agreed that the program met their expectations. The length of the intervention (six month) was rated as “optimal” by 6 participants, 4 rated it as “too short”. The frequency of the monitoring via mobile phone was rated as “optimal” by 9 out of 10 participants. Details on the evaluation of the intervention modules and ratings of the personal benefit are presented in Table 3.

### 3.6. Adherence and Program Use

In total 229 of 324 monitoring assessments were completed (70.7%). On average, participants of the intervention group completed 19.08 of maximum 27 monitoring questionnaires (SD = 8.31; range: 4–27) and 8 participants triggered 27 monitoring alerts (M = 3.38 per person; SD = 2.50; range: 1–9). Altogether, 27 alert phone calls were conducted to clarify if the participants were in need of further support. Overall, participants indicated in 85.2% of the completed monitoring assessments that they took their medication regularly as prescribed and 9 out of 12 participants indicated this in over 90% of the completed monitoring assessments. Moreover, participants stated in 87.8% of the completed assessments that they felt well or very well in the last week and in 83.8% that they slept more than 6 h per night on average. In 99.1% of the completed assessments, participants indicated that they had at least one social contact per day. The option to book chat appointments with the study clinician was not used by the participants at all and only one participant booked a telephone appointment.

### 3.7. Healthcare Utilization during Study Participation

Almost all participants (19 out of 20; 95.0%) assessed at T2 saw their psychiatrists nearly every four weeks during the 6 months of study participation (range: 1–13 sessions; M = 5.53, SD = 2.61). Only one participant in the control group did not see his/her psychiatrist. Moreover, 13 out of 20 participants (65.0%) received psychotherapeutic treatment (range: 1–27 sessions; M = 11.92, SD = 9.07). Six of these participants were in the intervention group and seven in the control group. Three participants in the control group were back in inpatient treatment during the study period (range: 3–91 days). Two patients (1 in each study group) were treated in day care hospitals (range: 7–9 weeks) and two patients (1 in each study group) had inpatient treatment in rehabilitation clinics (range: 7–9 weeks). One of these patients was treated in a day care hospital and in a rehabilitation clinic.

## 4. Discussion

The present study investigated the feasibility of an internet-based and mobile program aiming at the improvement of the continuity of care for individuals with schizophrenia spectrum disorders following their discharge from inpatient psychiatric treatment. During the course of the study it became apparent that recruiting the planned sample size was not feasible within a reasonable timeframe in a mono-center study with the described setup. While the overall number of patients with diagnoses within the schizophrenia spectrum who underwent inpatient psychiatric treatment was sufficiently high, an unexpectedly large proportion of these patients could not be enrolled in the trial.

Most patients could not be invited to participate because of the criteria regarding technical requirements, i.e., availability of internet access and possession of a mobile phone. This was the case even though we had ensured that participation would be possible with a simple mobile phone and would not require the possession of a smartphone. This finding confirms previous studies suggesting that the majority of patients in the schizophrenia spectrum have access to the internet and their own mobile phones, but by far not all patients may be reached by digital interventions: A meta-analysis from 2016 revealed an increase of mobile phone ownership among people with psychosis since 2007 and reported a rate of 81% for 2014/2015 [26]. Similarly, a study in the US reported that 73% of the sample (patients with schizophrenia) had access to a cell phone [27]. In 2019, a Spanish study reported a 94% rate of smartphone use in patients with early-stage schizophrenia [28]. Regarding internet access, the rates in individuals with schizophrenia spectrum disorders varied between 33% in Greece [29], 48% in the US [27] and 44% to 87% in Finland [29,30]. To our knowledge, no study investigated mobile phone ownership and internet access in German patients with schizophrenia spectrum disorders so far. Our results should remind researchers and clinicians to keep in mind the digital divide and the risk of excluding patients with severe mental illness from healthcare services due to their lack of access to technology [31].

Another factor that hindered recruitment in the present trial were organizational challenges, especially due to staff turnover and difficulties of the research team in tracking upcoming discharges and finding the right moment to invite patients to participate in the study before they left the clinic. These observations are equivalent to the ones recently reported by Allan et al. [32] who analyzed in detail barriers to recruitment in a digital intervention study for psychosis. Similar to the present study, these authors also reported that some patients could not be approached by the researchers because mental health professionals on the ward had decided in advance that patients would not be suitable for the study or capable of using the intervention. Thus, in future research it seems essential for the research team to motivate the clinical team well and to collaborate more closely during the recruitment process.

Aside from these aspects, a substantial proportion of invited patients declined study participation. The most commonly mentioned reason was that patients felt that they already had sufficient aftercare support. Also, more than half of the recruited sample received psychotherapeutic treatment during their study participation. This is an unexpectedly high percentage, given that previous studies reported very low rates of psychotherapeutic treatment in German patients with schizophrenia spectrum disorders (<1%) [33,34]. In combination with the fact that undergoing outpatient psychiatric treatment after discharge was an inclusion criterion, this means that patients invited to and included in the present RCT had access to comprehensive professional care which may have limited their interest in the digital intervention. In particular, due to past efforts of local treatment providers, psychotherapeutic care for individuals with psychosis is well developed in the Heidelberg region compared with other areas of Germany and internationally.

In addition, a subgroup of patients declined because they had no interest in the study, no time to participate, did not expect the intervention to be helpful or expected too much effort due to the study or the intervention, i.e., some patients might see such an intervention as an additional burden rather than a potential source of support. Patients with a high symptom level at discharge might be especially prone to decline additional support offers, because they might expect additional effort and do not feel that they have the capacity to spend it. Participating in a continuous intervention might also be disincentivizing for some patients as it underlines the necessity to include disease management constantly into daily life. Concerns about data privacy or feeling controlled by the program apparently played a minor role in declining participation. In order to overcome some of the above-mentioned challenges, future research should involve service users to a greater extent in the development of digital interventions. This could be a crucial step towards an enhanced understanding of how patients with schizophrenia spectrum disorders wish to be supported after discharge and how such interventions may be offered to them more successfully.

Patients who decided to participate in the present trial had overall positive attitudes towards the digital aftercare program and expected to benefit from it in terms of their well-being and feeling more secure and supported after discharge from the hospital. But even though most participants indicated that they would use counseling via internet chat or phone within the program, only one counseling appointment via phone was booked. However, 90% indicated in the post assessment that they like the idea that counseling appointments via internet chat and phone were offered. This leads us to the assumption that on the one side patients appreciate the opportunity to use these counseling options and that it might provide a feeling of security, while on the other side there might be a barrier to actually use it. Another explanation is that participants in the present study might have simply not needed additional counseling after discharge. This seems also plausible given that all participants were in psychiatric treatment and most also in psychotherapeutic treatment after discharge, seemed to feel quite well and showed a good medication adherence according to the monitoring data. Unlike the counseling module, the weekly supportive monitoring was used often and seemed to be very well accepted by the participants. This finding is in line with previous results on the acceptability of monitoring modules in other mental disorders (e.g., [35,36]) and in psychosis [37]. Overall, most participants were very satisfied with the digital intervention and indicated that they felt supported by the program. The results regarding attitudes and user satisfaction indicate that a mobile and internet-based intervention like HEINS might be a promising opportunity to provide aftercare support in schizophrenia spectrum disorders.

When it comes to the analyses of intervention effects, it must be clearly stated that the present study was not designed to evaluate the efficacy of the program. Moreover, the small sample size also did not allow a preliminary estimation of intervention effects. Overall, the mean PANSS scores at baseline in our study correspond to previous reports on the symptom status of patients with schizophrenia spectrum disorders at discharge (e.g. [38,39]) and suggest that the sample received effective inpatient treatment. The PANSS scores at post assessment were slightly lower compared with baseline and appeared to be similar in both study groups. The good symptom status of both groups, as well as the high number of patients in regular psychiatric and psychotherapeutic treatment, raise the question of how much room there might have been for intervention effects, but this would need to be addressed in further studies. Furthermore, the relatively short observation period of six months might have been too short to record potential worsening of symptoms in the control group and to show possible benefits of the intervention. Most other comparable studies covered at least 12 months [16,17,18,19]. But it is worth mentioning that three participants in the control group and none in the intervention group were re-hospitalized during the study period. However, the present study does not provide enough data to investigate associations with and effects on healthcare utilization.

## 5. Conclusions

The present RCT provides important insights and may inform other researchers about potential challenges in conducting such a study, and helps to minimize publication bias [40,41], even though it was not completed in accordance to the study plan. As digital interventions have been shown to be feasible and effective for various mental disorders including psychotic disorders [17,42], it seems reasonable to use the potential of such interventions for the improvement of the continuity of care for patients suffering from schizophrenia spectrum disorders. However, evidence on aftercare programs for schizophrenia spectrum disorders is still scarce. The present study provides insights into potential benefits of such a program but also points out potential barriers that need to be addressed by future research.

## Figures and Tables

**Table 1:** Demographic characteristics and clinical variables.

	**Intervention Group** (*N* = 12)	**Control Group** (*N* = 13)
Gender		
Female	66.7% (*N* = 8)	61.5% (*N* = 8)
Male	33.3% (*N* = 4)	38.5% (*N* = 5)
Mean age (SD)	34.3 (10.33)	38.2 (14.07)
Mean illness duration in years (SD)	7.6 (7.90)	8.0 (7.36)
Number of previous inpatient treatments due to the schizophrenia spectrum disorder	3.2 (3.83)	2.7 (3.47)
PANSS total score M (SD)		
Discharge from hospital (t1)	48.42 (16.63)	46.00 (12.49)
6-months assessment (t2)	44.30 (14.61)	43.30 (11.48)

*Note.* PANSS score t1: control group *N* = 11; t2: intervention group *N* = 10, control group *N* = 10.

**Table 2:** Attitudes and expectations.

**Would you use the following modules of HEINS?**	**Agreement**
Weekly monitoring	100.0% (*N* = 25)
Information materials	80.0% (*N* = 20)
Personal crisis plan	88.0% (*N* = 22)
Counseling via internet chat	72.0% (*N* = 18)
Counseling via phone	80.0% (*N* = 20)
Contact the hospital via HEINS in case of a crisis	92.0% (*N* = 23)
**How much do you agree?**	
I think that the program HEINS provides a feeling of security to the participants.	96.0% (*N* = 24)
I believe that participation in the HEINS program has a positive effect on one’s wellbeing.	92.0% (*N* = 23)
I think it’s good that one is called by a doctor in case of alarming answers in the monitoring via mobile phone.	92.0% (*N* = 23)
I think that I would benefit from the participation in HEINS.	88.0% (*N* = 22)
I think participants feel supported by the program HEINS after discharge.	88.0% (*N* = 22)
My motivation to participate in the HEINS program is high.	88.0% (*N* = 22)
I think that the weekly contact via monitoring helps participants to take their medications as prescribed.	80.0% (*N* = 20)
I think that participating in HEINS helps to cope with daily routine after discharge.	80.0% (*N* = 20)
I feel secure about the data privacy in the HEINS program.	75.0% (*N* = 18)
I am sufficiently supplied with aftercare support without the HEINS program.	72.0% (*N* = 18)
I think it’s good that the hospital offers an aftercare program like HEINS	80.0% (*N* = 20)
The effort to participate in the HEINS program seems low to me.	84.0% (*N* = 21)
In general, I have a positive attitude towards communication technologies (e.g., computer, mobile phone, internet).	84.0% (*N* = 21)

Note. *N* = 25. Answers on the 4-point Likert scale were dichotomized (Disagree: “definitely no”, “rather no”/“not at all”, “barely”; Agree: “rather yes”, “definitely yes”/“somewhat”, “very”).

**Table 3:** User Satisfaction.

	Agreement	Not Able to Evaluate
I like the idea that the program contains information materials on schizophrenia.	80.0% (*N* = 8)	10.0% (*N* = 1)
The information materials were helpful for me.	70.0% (*N* = 7)	10.0% (*N* = 1)
I like the idea of the weekly monitoring via mobile phone	100.0% (*N* = 10)	/
The monitoring feedback was appropriate	80.0% (*N* = 8)	/
The monitoring feedback was helpful for me.	60.0% (*N* = 6)	/
I like the idea that telephone and internet chat appointments are offered.	90.0% (*N* = 9)	10.0% (*N* = 1)
The utilization of telephone and internet chat appointments was helpful for me.	30.0% (*N* = 3)	50.0% (*N* = 5)
I like the idea that a personal crisis plan is prepared for every participant.	70.0% (*N* = 7)	30.0% (*N* = 3)
The personal crisis plan was helpful for me.	60.0% (*N* = 6)	20.0% (*N* = 2)
I felt supported by the HEINS program after discharge.	90.0% (*N* = 9)	
Participating in the HEINS program helped me to cope better with my daily routine after discharge.	70.0% (*N* = 7)	
Participating in the HEINS program had a positive effect on my wellbeing.	80.0% (*N* = 8)	
Participating in the HEINS program gave me a feeling of security.	60.0% (*N* = 6)	
Participating in the HEINS program supported me in dealing with my disorder.	60.0% (*N* = 6)	
Participating in the HEINS program helped me to take my medication as prescribed.	55.5% (*N* = 5)	

*Note*. *N* = 10. Answers on the 4-point Likert scale were dichotomized (Disagree: “applies not at all”, “applies barely”/“not at all”, “barely”; Agree: “applies somewhat”, “applies exactly”/“somewhat”, “very”). The last six items did not include the response option “not able to evaluate”.

## Data Availability

The data presented in this study are available on request from the corresponding author. The data are not publicly available due to legal and privacy issues.
